# Spatiotemporal Assembly of Bacterial and Fungal Communities of Seed-Seedling-Adult in Rice

**DOI:** 10.3389/fmicb.2021.708475

**Published:** 2021-08-05

**Authors:** Hyun Kim, Yong-Hwan Lee

**Affiliations:** ^1^Department of Agricultural Biotechnology, Seoul National University, Seoul, South Korea; ^2^Research Institute of Agriculture and Life Sciences, Seoul National University, Seoul, South Korea; ^3^Interdisciplinary Program in Agricultural Genomics, Seoul National University, Seoul, South Korea; ^4^Center for Fungal Genetic Resources, Seoul National University, Seoul, South Korea; ^5^Plant Genomics and Breeding Institute, Seoul National University, Seoul, South Korea; ^6^Plant Immunity Research Center, Seoul National University, Seoul, South Korea

**Keywords:** endophytic microbiota, niche differentiation, rice, vertical transmission, seedling growth

## Abstract

Seeds harbor not only genetic information about plants but also microbial communities affecting plants’ vigor. Knowledge on the movement and formation of seed microbial communities during plant development remains insufficient. Here, we address this knowledge gap by investigating endophytic bacterial and fungal communities of seeds, seedlings, and adult rice plants. We found that seed coats act as microbial niches for seed bacterial and fungal communities. The presence or absence of the seed coat affected taxonomic composition and diversity of bacterial and fungal communities associated with seeds and seedlings. Ordination analysis showed that niche differentiation between above- and belowground compartments leads to compositional differences in endophytic bacterial and fungal communities originating from seeds. Longitudinal tracking of the composition of microbial communities from field-grown rice revealed that bacterial and fungal communities originating from seeds persist in the leaf, stem, and root endospheres throughout the life cycle. Our study provides ecological insights into the assembly of the initial endophytic microbial communities of plants from seeds.

## Introduction

The relationship between a plant host and its microbiota begins in the seed, which has its own microbial community. The composition and diversity of the seed microbial community affects the vigor of seedlings and fitness of adult plants. For example, variations in seed microbiota contribute to the rate (Nelson, 2018; Rodríguez et al., 2020) and speed (Rochefort et al., 2019) of germination of plant seeds. The seed microbial communities also drive the successional assembly of environment-derived microbial communities in the root (Ridout et al., 2019). These effects of the seed microbial community on plant fitness provide opportunities for improving plant health. Previous studies have reported that compositional variations of the seed microbial community are affected by the geographical location where seeds are harvested (Barret et al., 2015), as well as by the harvesting year (Rochefort et al., 2019), seed compartment (Eyre et al., 2019), and plant domestication (Kim et al., 2020). Therefore, it is important to understand the variations in microbial communities associated with seeds.

Microbial communities associated with seeds were known to be distributed in the outer and inner space of seed coat, surface of grain, embryo, and endosperm (Eyre et al., 2019; Wang et al., 2020; Abdelfattah et al., 2021). A culture-dependent approach also reported that bacterial and fungal isolates are primarily retrieved from the seed coats of rice, barley, and alfalfa (Maude, 1996; Singh and Mathur, 2004). A previous study in rice reported that the seed coat (or husk) possesses higher richness and diversity of bacterial and fungal communities than grain does (Eyre et al., 2019). In particular, 85% of bacteria and 81% of fungi were found in the seed coat and the surface of the grain (Eyre et al., 2019). These findings indicate that the seed coat plays crucial roles in protecting genetic information of plants and microbes potentially associated with plants. Understanding on the effects of the seed coat on the assembly of microbial communities in developing seedlings will provide ecological importance of the seed coat in the plant–microbiome relationship.

Germination and seedling development are crucial steps in the life cycles of plants. The metabolic environment changes dynamically throughout seed germination and seedling growth (Bewley, 1997; He and Yang, 2013). Seed germination begins with imbibition (rehydration of seeds), which is a process of diffusion wherein water is absorbed by solid particles such as colloids (Bewley and Black, 1978). Upon imbibition, substrate, and energy starvation activate the embryo, and phytohormones (primarily gibberellic acid) are then produced. Gibberellic acid diffuses into the aleurone layer, leading to the synthesis of hydrolytic enzymes including α-amylases. These hydrolytic enzymes degrade storage compounds in the endosperm to support seedling establishment (He and Yang, 2013). Proteomic analysis showed sequential expression of protein sets involved in cell wall biosynthesis, as well as the assembly of mitochondria, biosynthesis of amino acids and starch, and aerobic respiration (He et al., 2011). Postgerminative growth of seedlings is primarily driven by cell expansion along the embryonic axis (shoot and root meristem) (Wolny et al., 2018). Transcriptional and hormonal regulation processes are involved in the early developmental stages of seedlings (Hoffmann-Benning and Kende, 1992; Magneschi and Perata, 2009; Pucciariello, 2020). Thus, the physiological changes that occur in germinating seeds and growing seedlings have been thoroughly examined from a biochemical perspective. However, little attention has been paid to the associated microbial communities.

One unsolved question regarding plant microbiomes is the origin of the microbial communities residing in seeds. Three putative transmission pathways, i.e., internal (via non-vascular or xylem tissues in maternal plants), floral (via the stigma of maternal plants), and external (transmitted from the external environment including air and soil), have been proposed (Maude, 1996; Nelson, 2018; Shahzad et al., 2018). Studies have reported the assembly of progeny seed microbial communities in this context. In bean and radish, bacterial communities could be transmitted via the floral pathway, as revealed in investigation of bacterial communities in reproductive organs (buds, flowers, and fruits) and seeds (Chesneau et al., 2020). In oilseed rape, the pollination activity of honeybee (*Apis mellifera*) affects progeny seed microbial communities by introducing insect-associated bacteria into flowers (Prado et al., 2020). Although the assembly of seed microbial communities has been examined, how these communities are transferred within internal tissues during plant development and colonize progeny seeds remains largely unknown.

In the present study, we describe the endophytic bacterial and fungal communities of the leaves, stems, and roots of rice plants grown in axenic culture and field conditions. The objectives of the study were: (1) to examine the temporal dynamics of seed bacterial and fungal communities during the germination and postgerminative growth of seedlings in axenic culture, (2) to identify vertical transmission of seed bacterial and fungal communities to progeny seeds, and (3) to reveal the distribution of vertically transmitted bacteria and fungi in rice endophytic tissues of field-grown rice.

## Materials and Methods

### Rice Cultivation in Axenic Cultures

To investigate the movement of seed-borne bacterial and fungal communities in rice seedlings grown under axenic conditions, surface-sterilized seeds were planted in 60 ml test tubes with 15 ml of Murashige and Skoog (MS) medium (4.4 g L^–1^ MS powder and 9 g L^–1^ agar powder; autoclaved at 121°C for 20 min). The test tubes were sealed with autoclaved silicone rubber stoppers and Parafilm to prevent entry of exogenous microbes. Rice seedlings were grown at 28°C and 80% humidity with 16-h light/8-h dark cycle in a growth chamber. Rice seedling samples were collected at 0, 1, 4, 7, and 14 days after planting. Three seedlings were collected at each sampling point. To exclude epiphytic fractions in the seedling samples, surface sterilization was performed. After surface sterilization, 14-day-old seedlings were divided into leaves, stems, and roots. Collected samples were stored in −80°C until DNA extraction. To compare the effects of seed coats on the bacterial and fungal communities of seedlings, hulled seeds (seeds without seed coats) were prepared using autoclaved forceps. Sample preparation of seedlings grown from hulled seeds was identical to of the process used for the intact seeds.

### Collection of Rice Samples Grown Under Field Conditions

To track the temporal changes in rice-associated microbial communities under field conditions parallelly with axenic cultures, rice plants were grown in a field located at the university farm of Seoul National University (37°16′06.7′′N 126°59′24.5′′E). For rice cultivation, a total of 9 kg nitrogen, 4.5 kg phosphate, and 5.7 kg potassium per 1,000 m^2^ was applied three times during the growing season. Sampling began 48 days after transplanting (tillering stage) into the field. Additional rice samples were collected at 90 (heading stage) and 141 (harvest) days after transplanting. The collected plant samples were divided into leaf, stem, and root portions. Roots were obtained using the method described by Edwards et al. (2015). The divided samples were surface-sterilized to remove epiphytic communities. The sterilized plant compartments were ground using sterilized mortars and pestles. 0.5 g of each ground sample was transferred to the Lysing Matrix E tube provided in the FastDNA SPIN Kit for Soil (MP Biomedicals, Solon, OH, United States). Seed samples were collected from the heading stage to harvest. The collected seeds were surface-sterilized and transferred to Lysing Matrix S tubes (MP Biomedicals, Solon, OH, United States). All samples were kept at −80°C until DNA extraction.

### Sample Preparation and DNA Extraction

DNA from all samples was extracted using the FastDNA SPIN Kit for Soil (MP Biomedicals, Solon, OH, United States). Plant tissues were prepared and pulverized using a bead beater (Biospec Products, Bartlesville, OK, United States) at 4,000 rpm for 2 min. This step was repeated after cooling in ice for 1 min. Plant DNA was extracted following the instructions of the manufacturer. The concentration of DNA samples was quantified using NanoDrop spectrophotometer (Thermo Fisher Scientific, Waltham, MA, United States). The extracted DNA was stored at −20°C until amplicons generation.

### Polymerase Chain Reaction Amplification and Sequencing

16S ribosomal RNA (rRNA) and internal transcribed spacer (ITS) amplicons were generated via a two-step polymerase chain reaction (PCR) amplification protocol. The V4 region of bacterial 16S rRNA gene was amplified with the universal PCR primers 515F and 806R (Caporaso et al., 2011). To reduce plant mitochondrial and plastid DNA contamination, peptide nucleic acid (PNA) PCR blockers were added during the first PCR step (Lundberg et al., 2013). The fungal ITS2 region of the 18S rRNA gene was amplified using ITS3 and ITS4 PCR primers (White et al., 1990). Each sample was amplified in triplicate in a 25-μl reaction tube containing 12.5 μl of 2 × PCR i-StarTaq^TM^ Master mix solution (iNtRON Biotechnology, Seongnam, South Korea), 0.4 μM each forward and reverse primers, 0.8 μM diluted DNA template and PNA clamps for chloroplast (pPNA) and mitochondria (mPNA), at 0.75 μM each. For generation of the ITS libraries, the conditions were the same except that PNA clamps were not included. PCR was performed using the following program: initial denaturing at 98°C for 3 min, followed by 32 cycles of denaturing at 98°C for 10 s, PNA annealing at 78°C for 10 s, primer annealing at 55°C for 30 s and extension at 72°C for 60 s. For ITS PCR amplification, the same program was used, but without the PNA annealing step. Each library was accompanied by negative PCR controls to ensure that the reagents were free of contaminant DNA. Amplicon replicates were pooled and then purified using the MEGAquick-spin Plus DNA Purification Kit (iNtRON Biotechnology, Seongnam, South Korea) with an additional ethanol clean-up step to remove unused PCR reagents and resulting primer dimers. Next, PCR was conducted with the Nextera XT Index Kit (Illumina, San Diego, CA, United States). DNA templates were diluted to equal concentrations after measurement with the Infinite 200 Pro (Tecan, Männedorf, Switzerland). The libraries were then pooled at equal concentrations into a single library and concentrated using AMPure beads (Beckman Coulter, Brea, CA, United States). The pooled library was then subjected to a final gel purification step to remove any remaining unwanted PCR products. Pooled libraries were sequenced using the Illumina MiSeq platform with 2 × 300 base pair read length. Sequencing was conducted at the National Instrumentation Center for Environmental Management (NICEM), Seoul National University, South Korea.

### Processing of Microbial Reads, and Statistical Analysis of Microbial Communities

The sequenced reads were processed with the QIIME2 (version 2018.6) pipeline (Callahan et al., 2016). After demultiplexing, the resulting sequences were merged using PEAR (Zhang et al., 2014) and then quality filtered with the DADA2 plugin in the QIIME2 (version 2018.6) pipeline (Callahan et al., 2016). High-quality sequences were clustered into operational taxonomic units (OTUs) using the open reference vsearch algorithm (vsearch cluster-features-open-reference) (Rognes et al., 2016) against the Silva 99% OTU representative sequence database (v132, April 2018) (Quast et al., 2012), and then assembled into an OTU table. Bacterial OTUs were filtered for chimeras using the vsearch uchime-*de novo* algorithm (Edgar et al., 2011). Fungal OTUs were checked for chimeric sequences using the Uchime-ref algorithm against the dedicated chimera detection ITS2 database (June 2017 version) (Nilsson et al., 2015). The taxonomy of non-chimeric OTUs was assigned using the Naïve Bayes algorithm implemented in the q2-feature-classifier prefitted to the Silva database for the V4 region of 16S rRNA genes (Bokulich et al., 2018). For the ITS2 region, taxonomic assignment was conducted with the q2-feature-classifier prefitted to the UNITE database (UNITE_ver7_dynamic of January 2017) (Nilsson et al., 2019). Bacterial sequences over 300 bp in length and fungal sequences shorter than 100 bp were discarded. The OTU table was imported into R software by the phyloseq package (McMurdie and Holmes, 2013) for further analysis. Sequences from host DNA and OTUs that were unassigned at the kingdom-level were removed (bacterial OTUs: orders “*Chloroplast*” and “*Rickettsiales*”; fungal OTUs: kingdoms “*Unassigned*,” “*Chromista*,” and “*Plantae*”).

### Statistical Analysis and Visualization

Unless otherwise noted, all statistical analyses were performed using R software (version 3.5.2) (R Core Team, 2013) and statistical significance was determined at *ɑ* = 0.05; where appropriate, the statistical significance was corrected for multiple hypothesis testing using the false discovery rate (FDR) method. The OTU table was normalized through cumulative-sum scaling (CSS) and log-transformed with the cumNorm function from the R package metagenomeSeq (v3.8) (Paulson et al., 2013). Rarefaction was assessed when calculating alpha diversity (McMurdie and Holmes, 2014). The Shannon and Simpson indices were calculated using the alpha function in the R package microbiome (v1.9.13) (Lahti and Shetty, 2019). The Kruskal–Wallis test and Dunn’s test were also performed in R. Taxa with relative abundances greater than 0.5% were visualized with the R package ggplot2 (v3.2.1) (Wickham, 2016) for taxonomic composition analysis. A Bray–Curtis dissimilarity matrix was constructed for principal coordinate analyses (PCoA) to compare community structure among examined samples based on both abundances and profiles of OTUs. Permutational multivariate analysis of variance (PERMANOVA) was conducted using the adonis function in the vegan package (Oksanen et al., 2018).

### Assessment of Movement of Seed Microbial Communities to Seedlings and Adult Plants

To assess the movement of microbial communities from seeds to seedlings, the community composition of germinating seeds and seedling compartments was compared to seed bacterial and fungal communities before planting. In this assessment, we assumed that community membership between seeds and other tissues is similar if seed OTUs are moved to the shoot, leaves, stems, and roots of seedlings and adult plants in the presence and absence concept. Since Jaccard dissimilarity is calculated by dividing the number of the shared OTUs between two communities by total numbers of OTUs (not consider OTU abundances), community dissimilarity was estimated using the Jaccard dissimilarity index using the R package vegan. OTU profiles of seeds and seedlings were compared using a Venn diagram to identify OTUs co-occurring among seeds prior to planting in MS agar medium and 7- and 14-day-old seedlings. For this analysis, Venn diagrams were constructed using InteractiVenn^1^ (Heberle et al., 2015). To investigate the movement of seed microbial communities to adult plants under field conditions, we first identified OTUs present in both seeds and rice compartments, including the leaf, stem, root, and progeny seeds. These OTUs were defined as seed-borne OTUs. Then, the proportion of seed-borne OTUs was calculated by dividing the number of seed-borne OTUs by the total number of OTUs in each compartment. The taxonomic composition of seed-borne OTUs was visualized using bar plots showing cumulative relative abundances.

## Results

### Composition of Bacterial and Fungal Communities in Rice Seedlings Grown in Axenic Cultures

We investigated the bacterial and fungal communities of rice seedlings grown in axenic culture (MS agar medium) to verify the microbial assembly present during the early growth of rice seedlings. Rice seedlings were grown from seeds with (unhulled condition) or without (hulled condition) seed coats. As the seedlings were grown in a germ-free medium, which prevents the input of microbes from the surrounding environment, the leaf, stem, and root endospheres of seedlings may harbor microbial communities originating solely from seeds. To completely exclude epiphytic microbes, additional surface sterilization was performed. Based on the sampling procedures, the collected samples were labeled using presence and absence of seed coats (H, hulled condition; U, unhulled condition), sampling point (0, prior to planting; 1, 1 day after planting; 4, 4 days after planting; 7, 7 days after planting; and 14, 14 days after planting), and plant compartment (Sh, shoot; L, leaf; S, stem; and R, root). As a result, 67,211 bacterial and 64,909 fungal reads were acquired in the hulled condition, and greater number of reads were obtained in the unhulled condition (251,968 bacterial and 300,500 fungal reads) (Supplementary Table 1).

**FIGURE 1:**
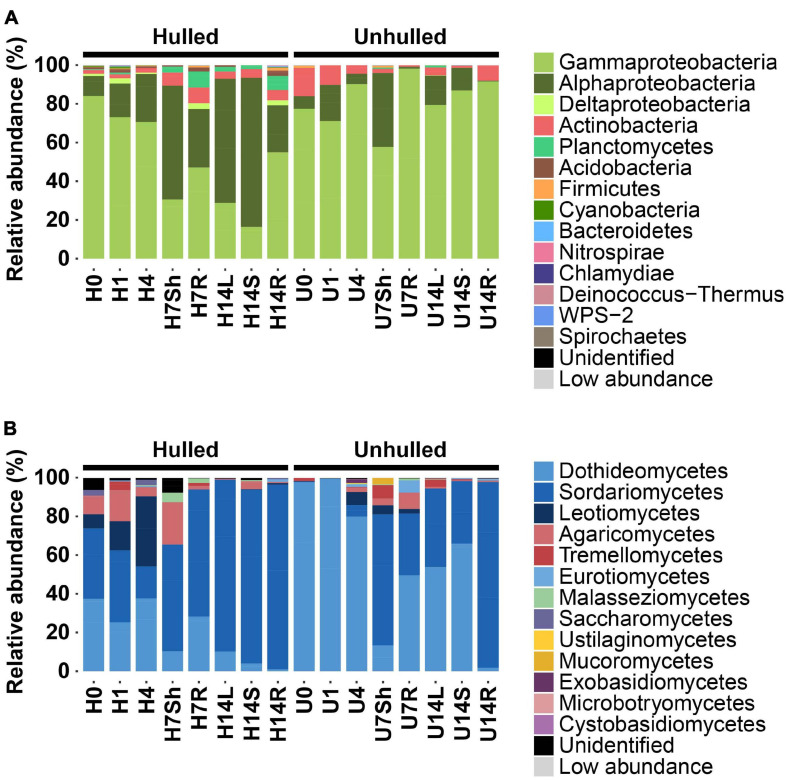
Abundance patterns of bacterial and fungal communities in seeds and seedlings. **(A)** Taxonomic composition of bacterial communities associated with seeds and seedlings grown from hulled and unhulled seeds. **(B)** Taxonomic composition of fungal communities associated with seeds and seedlings grown from hulled and unhulled seeds. In panels **(A,B)**, each bar indicates the taxonomic composition of a given sample. Colors indicate different phyla and classes in the bacterial and fungal communities. Low abundance refers to the group of phyla or classes with relative abundances lower than 0.05%. H and U indicate the absence and presence of seed coats, respectively. The numbers 0, 1, 4, 7, and 14 indicate the day(s) after planting on Murashige and Skoog (MS) agar medium. Sh, L, S, and R represent the endospheric regions of the shoots, leaves, stems, and roots, respectively. The exact numbers of sequence reads and relative abundances of samples are available in Supplementary Tables 1, 2.

The taxonomic composition of bacterial and fungal communities also differed between unhulled and hulled seeds (Figure 1). In the bacterial community, *Alphaproteobacteria* was dominant in the shoot (58.8%) and leaf endospheres (64.1%) of seedlings grown from hulled seeds, whereas *Gammaproteobacteria* was the most abundant class in unhulled seeds (Figure 1A and Supplementary Table 2; U0, 77.4%; U1, 71.1%; and U4, 90.1%) and their seedlings (U7Sh, 57.7%; U7R, 98.1%; U14L, 79.3%; U14S, 86.9%; and U14R, 91.5%). A similar pattern was observed in the fungal community (Figure 1B). In unhulled seeds, *Dothideomycetes* dominated the seed fungal community (Supplementary Table 2; U0, 97.6%; U1, 99.2%; and U4, 79.8%). Meanwhile, *Sordariomycetes* (H0, 36.4%; H1, 37.0%; and H4, 16.4%), *Leotiomycetes* (H0, 7.27%; H1, 15.0%; and H4, 36.2%), and *Agaricomycetes* (H0, 9.5%; H1, 16.1%; and H4, 4.7%) were dominant in hulled seeds. *Dothideomycetes* (U7Sh, 13.3%; U7R, 49.6%; U14L, 53.8%; U14S, 65.9%; and U14R, 1.8%) and *Sordariomycetes* (U7Sh, 67.7%; U7R, 31.8%; U14L, 40.4%; U14S, 32.3%; and U14R, 95.9%) were abundant in seedlings developed from unhulled seeds, whereas *Sordariomycetes* dominated the endospheric community of seedlings grown from hulled seeds (Supplementary Table 2; H7Sh, 55.1%; H7R, 65.7%; H14L, 88.7%; H14S, 89.8%; and H14R, 95.4%). These results indicate that seed coats provide ecological niches for the microbial communities. The compositional difference in seedling microbial communities in the presence vs. absence of seed coats suggests that the microbial pools present in seeds affect the endophytic microbial community composition of seedlings grown under axenic conditions.

### Effects of Seed Coat Presence, Compartment, and Age on Bacterial and Fungal Community Variations

**FIGURE 2:**
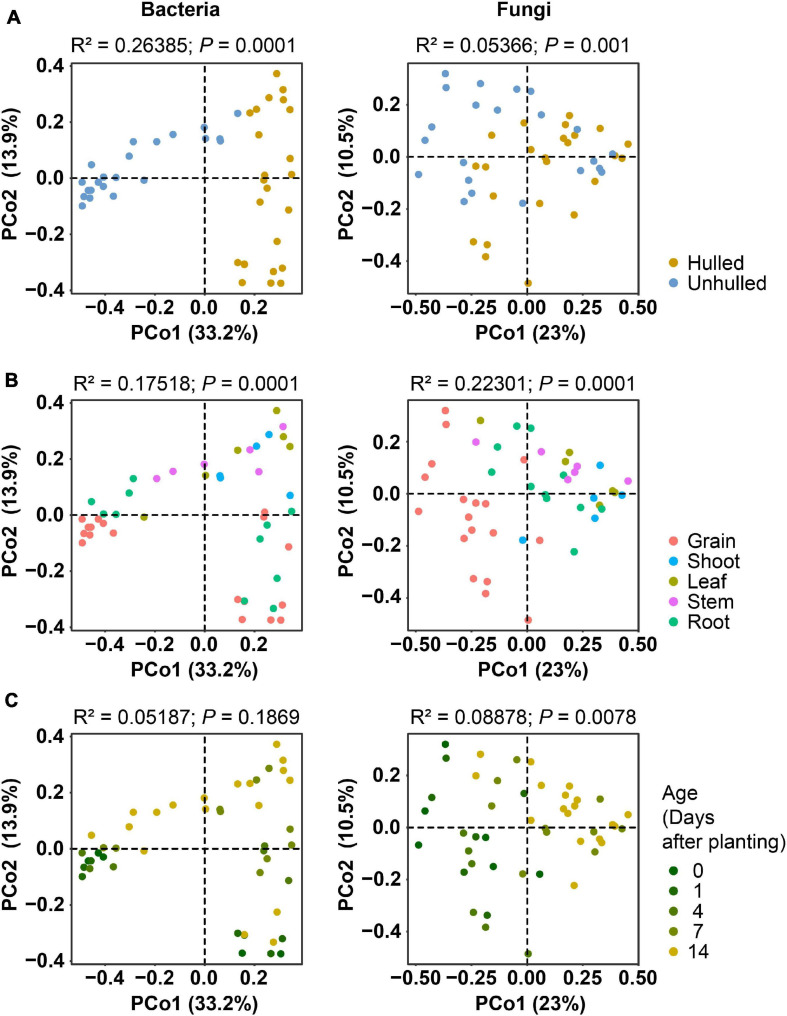
Ordination analyses of bacterial and fungal community compositions showing the effects of seed coat presence, plant compartment, and age. **(A)** Contribution of seed coats to compositional variations. The presence of seed coats (“Unhulled”) is indicated with blue dots, and the absence of seed coats (“Hulled”) is indicated with yellow dots. **(B)** Influence of rice compartments on compositional variations. Dots are colored according to compartment. **(C)** Effect of age on compositional variations. More vivid yellow dots denote older rice plants. In panels **(A–C)**, the ordination results for bacterial communities are displayed in the left panel, and those for fungal communities are shown in the right panel. Bacterial and fungal community distances were estimated using Bray–Curtis distances with cumulative sum scaling (CSS)-normalized and log-transformed operational taxonomic unit (OTU) abundances. Results on the permutational multivariate analysis of variance (PERMANOVA) are indicated on the top of each plot.

Next, we investigated the factors that contribute to compositional variations in bacterial and fungal communities during the early growth of rice seedlings. For this assessment, we employed ordination analysis (Figure 2). PCoA results showed that the bacterial communities cluster into two groups (“Hulled” and “Unhulled”) more clearly than fungal communities based on the presence or absence of the seed coat (Figure 2A). Bacterial and fungal communities were also grouped by compartment and age (Figures 2B,C). The effects of these factors on bacterial and fungal compositional variations were quantified using PERMANOVA. We found that the bacterial community (*R*^2^ = 0.26385, *P* = 0.0001) is more strongly affected by the presence of seed coats than the fungal community (*R*^2^ = 0.05366, *P* = 0.001) (Supplementary Table 3). Meanwhile, rice compartments contributed more to fungal compositional variations (*R*^2^ = 0.22301, *P* = 0.0001) than bacterial variations (*R*^2^ = 0.17518, *P* = 0.0001). On the other hand, age did not significantly affect bacterial compositional variations (*R*^2^ = 0.05187, *P* = 0.1869), while fungal community variations were significantly influenced by age (*R*^2^ = 0.08878, *P* = 0.0078).

Alpha diversity indices of bacterial and fungal communities also showed significant differences with compartment and age [Supplementary Figure 1; Kruskal–Wallis test, *P* = 0.00035 (bacterial richness); *P* = 0.01449 (fungal richness); *P* = 0.00137 (bacterial diversity); and *P* = 0.00103 (fungal diversity)]. In general, bacterial and fungal richness and diversity decreased under hulled conditions (Supplementary Figure 1). However, the bacterial richness and diversity of roots under hulled conditions decreased at 7 days after planting and increased thereafter. Similar patterns were observed for bacterial richness and diversity under unhulled conditions (Supplementary Figure 1). On the other hand, fungal richness and diversity increased in 4-day-old germinating seeds and decreased at 14 days after planting. These results suggest that microbial community composition and diversity are affected by the development of seedlings.

### Movement of Seed Microbial Communities Into Compartments of Seedlings

**FIGURE 3:**
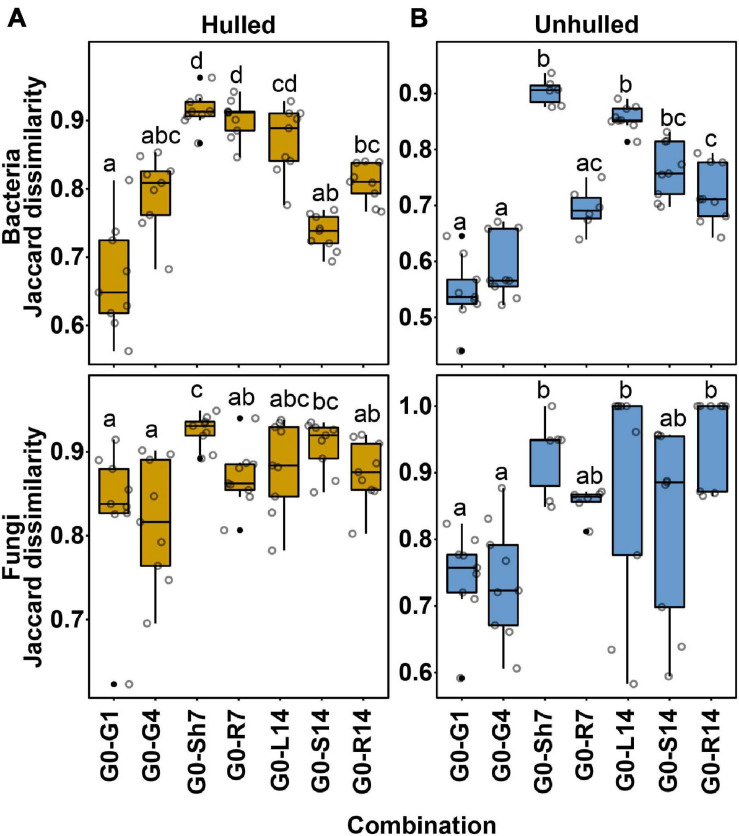
Community dissimilarity between microbial communities before and after planting. **(A)** Community dissimilarity in bacterial and fungal communities between samples taken before and after planting under hulled conditions. **(B)** Community dissimilarity in bacterial and fungal communities between samples taken before and after planting under unhulled conditions. Community dissimilarity was calculated using the Jaccard dissimilarity index. In panels **(A,B)**, the upper and lower panels show bacterial and fungal communities, respectively. Dots on each box plot indicate dissimilarity values estimated through pairwise comparison among biological replicates. Letters indicate the statistical significance, estimated using the Kruskal–Wallis test followed by Dunn’s test. The results of the statistical analysis are available in Supplementary Table 4. G0, seed microbial community before planting; G1, seed microbial community at 1 day after planting; G4, seed microbial community at 4 days after planting; Sh7, microbial community of the shoot endosphere at 7 days after planting; R7, microbial community of the root endosphere at 7 days after planting; L14, microbial community of the leaf endosphere at 14 days after planting; S14, microbial community of the stem endosphere at 14 days after planting; and R14, microbial community of the root endosphere at 14 days after planting.

We examined whether seed-borne OTUs are transmitted to the endosphere of the resulting seedlings. For this assessment, OTU profiles were compared between the microbial communities of seeds at the sowing stage (G0) and compartments of seedlings at 7 and 14 days after planting. We assumed that bacterial and fungal communities of seeds and seedlings consist of similar microbial members if seed-borne OTUs are moved to the compartments of developing seedlings. Dissimilarity of community membership was quantified using the Jaccard distance (*D* = 1−Jaccard similarity). *D* values near 0 indicate that the two microbial communities share a large number of OTUs. In bacterial communities, the Jaccard distances between seeds and germinating seeds or seedlings ranged from 0.5623 to 0.9627 for the hulled condition and from 0.4401 to 0.9367 for the unhulled condition (Supplementary Table 4). Meanwhile, the Jaccard distances of the fungal communities ranged from 0.6227 to 0.9492 for the hulled condition and from 0.5829 to 1 for the unhulled condition (Supplementary Table 4). The bacterial community composition of seeds at 1 day (Hulled, *D* = 0.6683 ± 0.078; Unhulled, *D* = 0.5463 ± 0.059) and 4 days (Hulled, *D* = 0.7943 ± 0.054; Unhulled, *D* = 0.5887 ± 0.057) after planting differed less from that of seeds at the sowing stage under both hulled and unhulled conditions compared to other compartments (Figure 3; Supplementary Table 4). The compositional difference increased with time and the differentiation of plant parts. For example, the endophytic bacterial communities of 7-day-old shoots (Hulled, *D* = 0.8702 ± 0.005; Unhulled, *D* = 0.9032 ± 0.023) and 14-day-old leaves (Hulled, *D* = 0.8992 ± 0.029; Unhulled, *D* = 0.8564 ± 0.022) showed large differences compared to the seeds at the sowing stage (Figure 3 and Supplementary Table 4). This tendency was also observed for fungal communities, although the differences were less significant than those observed for bacterial communities. This finding suggests that the differentiation and growth of plant tissues may lead to differentiation of the endophytic microbial communities.

**FIGURE 4:**
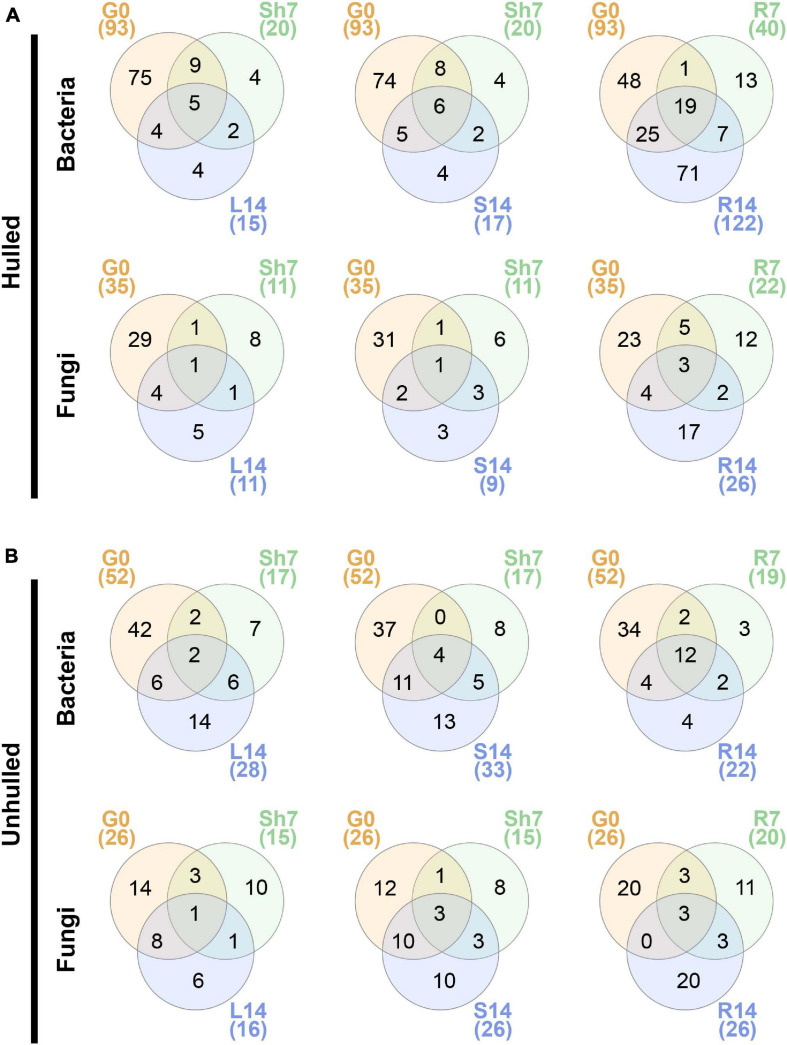
Distribution of bacterial and fungal OTUs in seeds and seedling compartments. **(A)** Distribution of bacterial and fungal OTUs in hulled seeds and their seedlings. **(B)** Distribution of bacterial and fungal OTUs in unhulled seeds and their seedlings. Venn diagrams were constructed using InteractiVenn (http://www.interactivenn.net/). Each circle in the Venn diagrams represents a given sample. The numbers in parentheses indicate the total numbers of OTUs detected in each sample. The numbers on the Venn diagrams indicate the numbers of OTUs uniquely detected or co-occurring across different samples. Taxonomic information for OTUs co-occurring in seeds, 7-day-old seedlings, and 14-day-old seedlings is available in Supplementary Table 5. G0, seed microbial community before planting; Sh7, microbial community of the shoot endosphere at 7 days after planting; R7, microbial community of the root endosphere at 7 days after planting; L14, microbial community of the leaf endosphere at 14 days after planting; S14, microbial community of the stem endosphere at 14 days after planting; and R14, microbial community of the root endosphere at 14 days after planting.

Next, we examined the distribution of OTUs in seeds at the sowing stage and each compartment of 7- and 14-day-old seedlings under hulled and unhulled conditions. We found that limited fractions of the seed microbial communities could be moved to both above- and belowground compartments. For example, 2–6 bacterial OTUs and 1–3 fungal OTUs co-occurred in the seeds and aboveground compartments of 7- and 14-day-old seedlings (Figure 4). These OTUs belonged to the bacterial genera *Burkholderia*, *Ralstonia*, *Sphingomonas*, and *Bradyrhizobium* and the fungal genus *Pyricularia* in hulled seeds and their seedlings (Supplementary Table 5). On the other hand, in unhulled seeds and their seedlings, co-occurring OTUs were assigned to the bacterial genera *Pantoea* and *Sphingomonas*, the fungal genus *Cladosporium*, and fungal family *Didymellaceae*. Greater numbers of seed OTUs were shared with root endosphere of seedlings than with other compartments. Those shared OTUs belonged to the bacterial genera *Burkholeria*, *Sphingomonas*, *Leifsonia*, *Aquabacterium* (in the hulled condition), *Pseudomonas*, *Pantoea*, *Methylobacterium*, *Curtobacterium* (in the unhulled condition) and the fungal genera *Pyricularia*, *Cladosporium*, and *Alternaria*. Generally, bacterial OTUs co-occurring between seeds and the stem endosphere of 14-day-old seedlings (Hulled, 64.7%; Unhulled, 45.4%) were more shared than the leaf endosphere of seedlings of the same age (Hulled, 60%; Unhulled, 28.5%). In contrast, in the fungal community, more OTUs were shared between seeds and the leaf endosphere (Hulled, 45.4%; Unhulled, 56.2%) than stem endosphere (Hulled, 33.3%; Unhulled, 50%).

### Movement of Seed Bacterial and Fungal Communities Under Field Conditions

**FIGURE 5:**
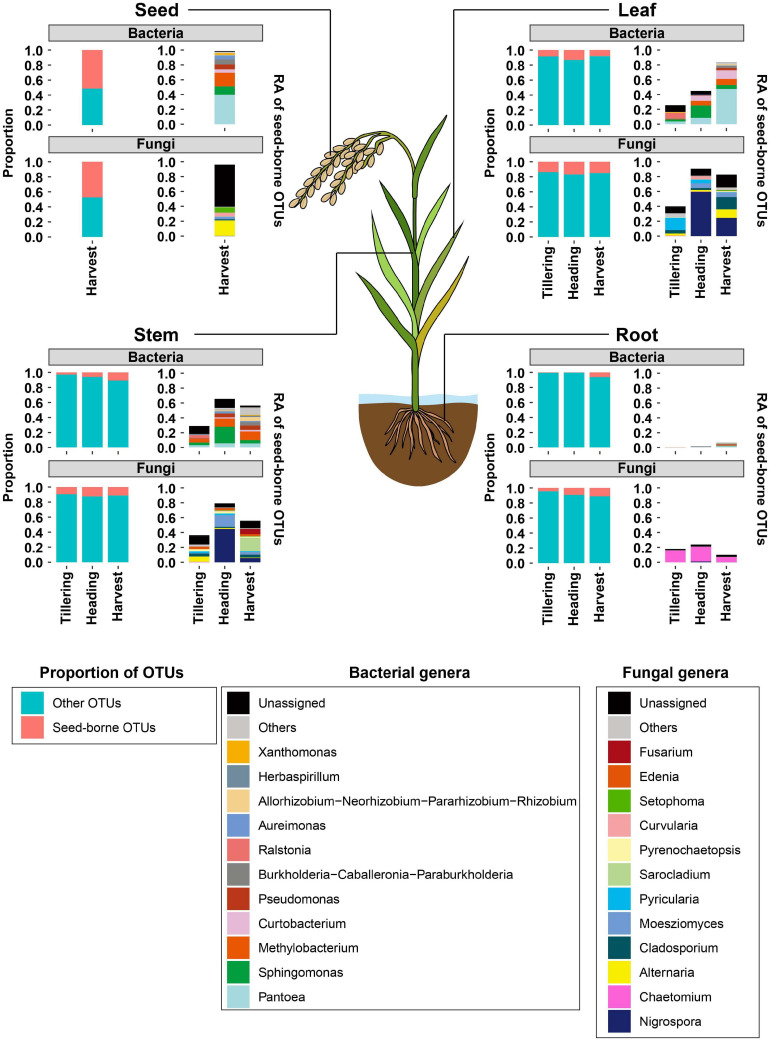
Distribution of seed-borne bacterial and fungal communities in compartments of field-grown rice plants. The distributions of seed-borne bacterial and fungal OTUs were investigated at three developmental stages: tillering, heading, and harvest. At each developmental stage, the proportion of seed-borne OTUs in the leaf, stem, root, and seed endospheres was calculated as the number of seed-borne OTUs divided by the total numbers of OTUs in that compartment. The proportions of seed-borne OTUs are indicated with magenta-colored bars. The remaining portions are indicated with cyan-colored bars. Taxonomic composition of seed-borne OTUs in plant compartments is shown at the genus level. Each color on the bar plots represents a bacterial or fungal genus. Genera with relative abundance lower than 0.03 are grouped into “Others.” The raw data on the relative abundances of bacterial and fungal genera are available in Supplementary Table 6. RA, relative abundance.

Since the movement of seed microbial communities from the seed to seedling endosphere had been observed under *in vitro* conditions, we investigated whether seed microbial communities can be moved to and colonize the rice leaf, stem, root, and progeny seeds during rice growth under field conditions. For this test, the distributions of seed-borne microbial communities in leaves, stems, roots, and progeny seeds collected from field-grown rice plants were examined. First, we identified seed-borne OTUs present at the sowing stage (0 days after transplanting) and in other compartments at the tillering, heading, and harvest stages. Almost half of bacterial (51.4%) and fungal (47.3%) OTUs co-occurred in seeds at the sowing and harvest stages (Figure 5 and Table 1). The proportions of seed-borne bacterial and fungal OTUs were lowest at the tillering stage, and increased throughout rice growth in the leaf, stem, and root endospheres (Figure 5 and Table 1). The proportion of seed-borne OTUs was small but accounted for the vast majority of relative abundances of bacterial and fungal communities. In progeny seeds, seed-borne OTUs accounted for 98.6 and 96.1% of relative abundances of the bacterial and fungal communities, respectively (Figure 5, Table 1, and Supplementary Table 6). Meanwhile, aboveground (leaf and stem) and belowground compartments exhibited differing patterns of seed-borne OTU abundances. Among the aboveground compartments, seed-borne OTUs in the leaf endosphere accounted for 25.7% (bacteria) and 40% (fungi) of relative abundances of microbial communities at the tillering stage. In the stem, 28.9% (bacteria) and 36.4% (fungi) of relative abundances were seed-borne OTUs at the tillering stage. During rice development, the cumulative relative abundances of seed-borne OTUs increased to 83.2% (leaf bacterial community), 56.5% (stem bacterial community), 82.5% (leaf fungal community), and 55.9% (stem fungal community) (Figure 5, Table 1, and Supplementary Table 6). On the other hand, seed-borne OTUs in the root endosphere were less dominant in the bacterial (tillering, 0.4%; heading, 1.3%; and harvest, 6.3%) and fungal (tillering, 17.8%; heading, 23.7%; and harvest, 9.8%) communities compared to aboveground compartments at the same developmental stages. This suggests that seed-borne OTUs are dominant in aboveground compartments, whereas the majority of root microbial communities originate from the soil environment under field conditions.

**TABLE 1:** Seed-borne bacterial and fungal operational taxonomic units (OTUs) in the leaf, stem, root, and progeny seeds.

Kingdom	Compartment	Stage	Number of seed-borne OTUs	Total number of OTUs	Proportion of seed-borne OTUs	Cumulative abundance of seed-borne OTUs
Bacteria	Seed	Harvest	36	70	0.5143	0.986
		Tillering	11	127	0.0866	0.257
	Leaf	Heading	22	166	0.1325	0.449
		Harvest	49	588	0.0833	0.832
		Tillering	25	855	0.0292	0.289
	Stem	Heading	51	843	0.0605	0.654
		Harvest	65	601	0.1082	0.565
		Tillering	7	1,116	0.0063	0.00427
	Root	Heading	6	1,356	0.0044	0.0136
		Harvest	27	436	0.0619	0.0633
Fungi	Seed	Harvest	63	133	0.4737	0.961
		Tillering	39	285	0.1368	0.4
	Leaf	Heading	54	317	0.1703	0.907
		Harvest	83	552	0.1504	0.825
		Tillering	58	613	0.0946	0.364
	Stem	Heading	81	653	0.1240	0.792
		Harvest	84	754	0.1114	0.559
		Tillering	10	233	0.0429	0.178
	Root	Heading	18	195	0.0923	0.237
		Harvest	19	171	0.1111	0.0984

When focusing on the taxonomic composition of seed-borne OTUs, *Pantoea*, *Sphingomonas*, *Methylobacterium*, *Curtobacterium*, and *Pseudomonas* were dominant in the bacterial communities of aboveground compartments (Figure 5 and Supplementary Table 7). Meanwhile, in fungal communities, *Nigrospora*, *Alternaria*, *Sarocladium*, *Cladosporium*, and *Moesziomyces* were identified as seed-borne OTUs under field conditions (Figure 5 and Supplementary Table 7). The bacterial genera *Pantoea*, *Ralstonia*, *Methylobacterium*, *Allorhizobium*-*Neorhizobium*-*Pararhizobium*-*Rhizobium*, *Burkholderia*-*Caballeronia*-*Paraburkholderia*, *Herbaspirillum*, and the fungal genera *Chaetomium*, *Alternaria*, *Cladosporium*, *Pyrenochaetopsis*, and *Setophoma* were identified as seed-borne taxa present in root microbial communities (Figure 5 and Supplementary Table 7). Among these taxa, we aimed to identify the specific bacterial and fungal taxa that could move to seedling compartments under axenic conditions and were also present under field conditions. *Pantoea*, *Methylobacterium*, and *Sphingomonas* were identified in the bacterial communities of both the leaf and stem endospheres under axenic and field conditions (Supplementary Tables 5, 7). In the fungal community, *Nigrospora*, *Alternaria*, *Malassezia*, Unidentified genus belonging to *Didymellaceae*, and *Pyricularia* were seed-borne OTUs in aboveground compartments under both axenic and field conditions (Supplementary Tables 5, 7). These common OTUs suggest that seed-borne OTUs may outcompete environmentally transmitted microbial communities.

## Discussion

The seed coat is a protective layer, as well as a channel for transmitting environmental cues to the interior of the seed (Radchuk and Borisjuk, 2014). The seed coat could also act as an ecological niche to support microbial communities. We found that the compositions of the microbial communities associated with seeds and seedlings differed according to the presence of the seed coats (Figures 1, 2). This is corroborated by the study reporting that seed compartments (outer husk, husk, outer grain, and grain) harbor different bacterial and fungal community compositions (Eyre et al., 2019). Compositional differences between hulled and unhulled seeds were also detected in seedlings grown under axenic conditions. This finding suggests that endophytic microbial communities in emerging tissues are shaped by endogenous seed microbial pools under axenic conditions.

In the present study, we investigated movement of seed-borne OTUs to seedlings and adult plants under axenic and field conditions. We found that bacterial OTUs belonging to *Pantoea*, *Sphingomonas*, and *Methylobacterium* and fungal OTUs belonging to *Alternaria*, *Cladosporium*, and *Pycularia* were commonly found as seed-borne taxa able to colonize in leaf and stem endosphere under axenic and field conditions (Figure 5 and Supplementary Table 5). However, the distribution patterns of seed-borne OTUs showed differences between axenic and field conditions in root endosphere communities. In the axenic culture, about 50% of bacterial and fungal OTUs co-occurred in the root endosphere and seeds (Figure 4), whereas the proportion and cumulative relative abundance of seed-borne OTUs were lower in the root endospheres of field-grown rice (Figure 5). These findings suggest that root bacterial and fungal communities originating from seeds might be depleted through the competition with invaders from the soil. Meanwhile, at the late stage of life cycle of rice under field condition, these seed-borne taxa dominated the endophytic communities of leaf and stem, suggesting that seed-borne microbial communities colonizing in the endosphere of the aboveground compartments could stand competition with external microbes.

Ordination and community dissimilarity analyses revealed that both bacterial and fungal compositions differed by compartment (above- and belowground tissues) and age (Figures 2, 3). In particular, compartment was the significant factor shaping bacterial and fungal community compositions. This relationship could be driven by a combination of niche differentiation due to temporal changes in plant physiological conditions, plant regulatory cues limiting microbial transmission, and microbial traits (Abdelfattah et al., 2021). Previous studies reported physiological and metabolic differences between the leaves and roots of wheat (Kang et al., 2019), rice (Baldoni et al., 2016), and barley (Klem et al., 2019). Morphological differences, photosynthesis and chlorophyll metabolism, and differential distributions of metabolites (including amino acids and carbohydrates) lead to niche differentiation between above- and belowground compartments during plant development. Parallel and longitudinal investigation of the transcriptome, proteome, metabolome, and microbiome in plant tissues will reveal the molecular mechanisms underlying niche differentiation and their relationships with plant microbiomes.

The diversity of bacterial and fungal communities was affected by the development and growth of seedlings (Supplementary Figure 1). A decrease in microbial diversity during seedling growth was reported for 28 plant genotypes belonging to Brassicaceae (Barret et al., 2015). During the germination and development of seeds and seedlings, the chemical properties of the seeds and surrounding environment change dramatically due to exudation (Bewley, 1997; Higashinakasu et al., 2004). Seed exudates contain carbohydrates, organic acids, fatty acids, amino acids, proteins, and other secondary metabolites (Schiltz et al., 2015). This finding indicates that seed exudates could cause the surrounding environment to become copiotrophic. The exudates of germinating seeds may affect distribution of oligotrophs and copiotrophs and may reduce microbial diversity similar to the effects of nutrient addition on soil microbial communities (Leff et al., 2015; Zeng et al., 2016). After germination, the changes in microbial richness and diversity differed according to the presence of seed coats and the kingdom (Supplementary Figure 1). An investigation of viable seed microbial communities and their correlations with seed exudates could provide more information about the effects of plant physiology on seed-borne microbial communities.

We identified not only OTUs shared among seeds and compartments of developing seedlings but also OTUs uniquely distributed in seeds or developing seedlings under axenic conditions (Figure 4). The compositional differences between microbial communities of seeds and compartments of seedlings might be related to intrinsic variability in seed microbial communities and seed rare taxa which abundances are below detection level in seeds but can proliferate in developing seedlings. The variability of microbial communities within seed populations have been reported in maize and common beans (Rosenblueth et al., 2012) and wheat (Özkurt et al., 2020). This seed-to-seed variability of microbial communities may affect compositional variability of developing seedlings. The similar results were also reported in acorn (Abdelfattah et al., 2021). Experimentally, test tubes where seedlings were grown were capped by a silicone rubber stopper that is widely used for microbial cultures. We also used a MS agar medium on which both plants and microbes can grow. We did not observe visible bacterial colonies and fungal mycelia which can be considered as contamination on the MS agar media during the seedling growth. Based on this observation, we considered that external contaminations are blocked during the experiment. Although we could not specify the reasons for the existence of unique OTUs in the developing seedlings, our findings suggest that seed bacterial and fungal communities could move from seed to developing seedlings partially.

We revealed that seed-borne OTUs in both bacterial and fungal communities are the main colonizers of the endospheres of leaves and stems (Figure 5). Movement of these OTUs occurred during growth of seedlings (Figure 4). Previous research reported that 45% of the bacterial community of first generation are vertically transmitted to seeds of second generation (Hardoim et al., 2012). We also found that 51.4% of bacterial OTUs and 47.3% of fungal OTUs were inherited by second-generation seeds. *Pantoea*, *Methylobacterium*, *Pseudomonas*, and *Sphingomonas* were commonly identified among the inherited taxa. These taxa were also identified in the seeds of wild species and their relatives in the genus *Oryza* (Kim et al., 2020). Seeds of radish (Rezki et al., 2018), bean (Chesneau et al., 2020), and maize (Johnston-Monje and Raizada, 2011) also harbor *Pantoea* and *Pseudomonas*, suggesting that these taxa may be preserved in the seeds of many plant species. These taxa may persist across generations in radish via the floral pathway, in which microbes are transmitted via reproductive organs (Chesneau et al., 2020). This finding implies that *Pantoea* and *Pseudomonas* may be vertically transmitted through both internal and floral pathways.

We found that fungal communities in rice seeds and seedlings are dominated by *Nigrospora*, *Pyricularia*, *Alternaria*, and *Cladosporium.* While seed fungal communities are less studied than bacterial communities, there have been a few reports that *Alternaria*, *Cladosporium*, and *Pleosporaceae* are abundant in the seed fungal communities in rice cultivars and wild rice species (Eyre et al., 2019; Kim et al., 2020), as well as in plants belonging to *Brassicaceae* (Barret et al., 2015), suggesting that ecological niches in seeds might be suitable for these fungal genera regardless of plant species. Similar to bacterial communities, we observed that fungal communities could be vertically transmitted through the seed. OTUs belonging to *Nigrospora*, *Alternaria*, *Cladosporium*, and *Moesziomyces* could persist throughout the growing season (Figure 5 and Supplementary Table 7). These OTUs were distributed in the leaf and stem compartments (Figure 5 and Supplementary Table 7). In forbs, which are herbaceous dicot plants, *Alternaria* and *Cladosporium* can be vertically transmitted and occur in seeds, cotyledons, and true leaves (Hodgson et al., 2014). Fungal taxa abundant in seeds were less common in the roots and rhizosphere of the common sunflower (*Helianthus annuus*) (Leff et al., 2017). The findings of the present study and previous reports suggest that fungi capable of vertical transmission might proliferate in ecological niches of the endosphere of aboveground compartments. Future works similar to the previous study on *Epichloë*, which demonstrated systemic colonization of in stem endosphere by the fungus during the growth of grasses (Kayano et al., 2018), will provide the evidence for vertical transmission of fungal community members via aboveground tissues.

## Conclusion

The present study provides novel insights into the dynamics of seed microbial communities during early developmental stages and seed maturation. We conducted temporal analyses of the bacterial and fungal communities associated with rice seeds and adult plants using amplicon-based community profiling. Through this approach, we found that seed bacterial and fungal communities could move from seeds to above- (shoots, leaves, and stems) and belowground compartments of seedlings. We also identified bacterial and fungal OTUs that were vertically transmitted and systemically distributed in aboveground compartments. The next step is to assess the functional properties of microbial communities and identify host factors, including genes, phytohormones, and metabolites, affecting their distribution in germinating seeds, seedlings, and adult plants over time. This approach will provide comprehensive insights into the temporal shift in ecological niches caused by host factors, as well as microbial functions that affect host physiology. This study provides an ecological basis for understanding the establishment of seed-borne plant microbiomes.

## Data Availability Statement

The datasets presented in this study can be found in online repositories. The names of the repository/repositories and accession number(s) can be found below: https://www.ncbi.nlm.nih.gov/, PRJNA728672 and PRJNA733292. Raw input files, and all the codes used for statistical analyses in this study are available at https://github.com/hyunkim90/seed_to_seedling_movement.

## Author Contributions

HK and Y-HL conceived and designed the study, discussed and interpreted the results, and contributed to the writing of the manuscript. HK carried out all experiments and analyzed the data. Both authors read and approved the final manuscript.

## Conflict of Interest

The authors declare that the research was conducted in the absence of any commercial or financial relationships that could be construed as a potential conflict of interest.

## Publisher’s Note

All claims expressed in this article are solely those of the authors and do not necessarily represent those of their affiliated organizations, or those of the publisher, the editors and the reviewers. Any product that may be evaluated in this article, or claim that may be made by its manufacturer, is not guaranteed or endorsed by the publisher.

## Footnotes

1
http://www.interactivenn.net/
