# Supplementary material for: Spatiotemporal Assembly of Bacterial and Fungal Communities of Seed-Seedling-Adult in Rice
Source: Front Microbiol. 2021 Aug 5;12:708475. doi: 10.3389/fmicb.2021.708475 (PMC8375405; doi:10.3389/fmicb.2021.708475)
Supplement: Supplementary file 1 [file Data_Sheet_1.DOCX]

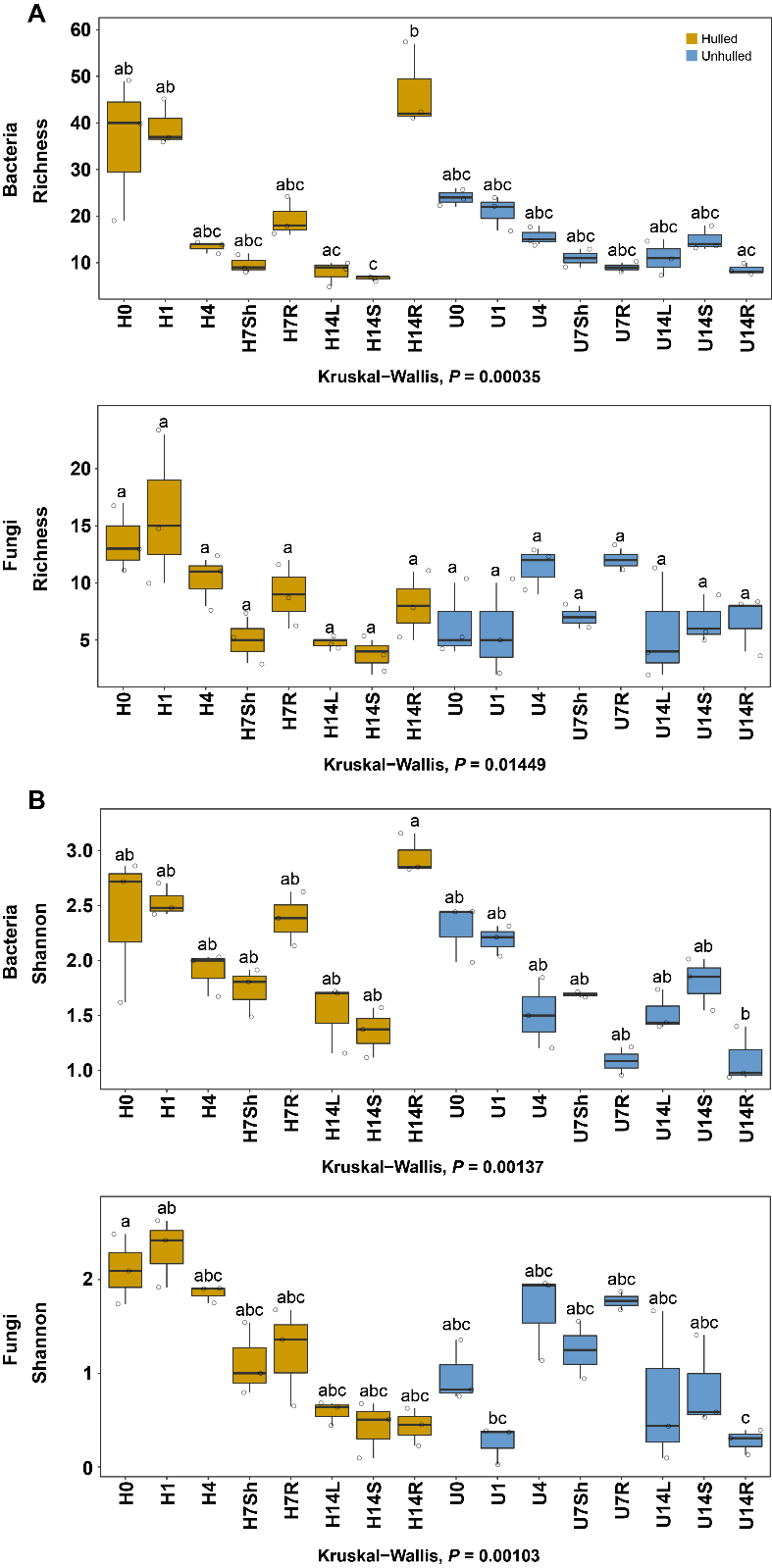


**Supplementary Figure 1. Alpha diversity of bacterial and fungal communities of seeds and seedlings.** (**A**) Richness of bacterial and fungal communities of seeds and seedlings. (**B**) Shannon index of bacterial and fungal communities of seeds and seedlings. In the panels **A** and **B**, the upper and lower panels show bacterial and fungal communities, respectively. Dots in the box plots indicate the diversity values of biological replications of each sample. The colors of boxes indicate the presence (“Unhulled”, blue) and absence (“Hulled”, yellow) of seed coats. The letters indicate the statistical significance estimated via Kruskal-Wallis test followed by Dunn’s test. H and U indicate the absence and presence of seed coats, respectively. The numbers 0, 1, 4, 7, and 14 mean the day(s) after planting on MS agar medium. Sh, L, S, and R represent the endospheric regions of shoot, leaves, stems, and roots, respectively.
